# Follicular Thyroid Carcinoma in a Country of Endemic Iodine Deficiency (1994–2013)

**DOI:** 10.1155/2018/6516035

**Published:** 2018-02-25

**Authors:** Edmund Muonir Der

**Affiliations:** ^1^Department of Pathology, Korle-Bu Teaching Hospital, P.O. Box 77, Korle-Bu, Accra, Ghana; ^2^Department of Pathology, University for Development Studies, P.O. Box 1883, Tamale, Ghana

## Abstract

**Background:**

Follicular thyroid cancer (FTC) has historically been linked to iodine deficiency. Although Ghana is among the iodine deficient regions of the world, the proportions, trends, and the clinical features of FTCs have not been studied as a single disease entity. The aim of this study was to determine the relative frequencies, trends, and the clinicopathological characteristics of FTCs among all thyroid malignancies in our institution.

**Materials and Methods:**

This was a retrospective study from January 1994 to December 2013. Data were analysed using SPSS software version 23 (Chicago) and Graph pad prism version 5.00.

**Results:**

Follicular thyroid cancer was the second thyroid malignancy (35.0%) and showed a gradual rise in relative proportions over the period. The male-female ratio was 1 : 1.5. The mean ages were 46.9 (SD ±17.3) for males and 46.4 (SD ±13.3) years for females. Enlarged palpable anterior neck swelling was the commonest symptom in males (86.7%) and females (91.3%) (*P* = 0.730). Hurthle cell carcinoma was the commonest variant of FTC, with 26.7% males and 10.6% females (*P* = 0.116). Distant spread was found in 23.3% of males compared to 19.1% of females (*P* = 0.633). The common sites of distant spread were bones (57.2%) in males and cervical lymph nodes (44.4%) in females (*P* = 0.106).

**Conclusion:**

Follicular thyroid cancer was the second common thyroid malignancy (35.0%) with a gradual rise in trend over the study period and male-female ratio of 1.5 : 1. Large anterior neck swelling was the commonest clinical presentation of FTC.

## 1. Introduction

Follicular thyroid carcinoma (FTC) is a malignant epithelial tumour showing follicular cell differentiation and lacking the diagnostic nuclear features of papillary thyroid carcinoma (PTC) [1]. The incidence of FTCs varies across the globe reflecting the population studied, the environmental factors, the method of study, and the iodine status of the area [2]. Studies in Ghana [3, 4], some parts of Africa [5, 6], and beyond [7, 8] have found FTC as the second common type of differentiated carcinomas of the thyroid gland. However, others based on the low levels of iodine in certain geographical location [9, 10] found it to be the commonest differentiated carcinoma of the thyroid gland [11–13]. Follicular carcinoma usually presents as a single palpable neck mass. Incidence is 13–17% of all thyroid carcinomas [14]. It is common in middle and older age groups; the average age at diagnosis is 50 years. It affects females more commonly than males [14–19]. Follicular thyroid cancer has historically been linked to iodine deficiency. The proportions, trend, and the clinical features of FTCs in Ghana, an iodine deficient country [20–22], have not been studied as a single entity. The aim of this was to determine the relative frequencies, trends, and the clinicopathological characteristics of FTC among thyroid malignancies diagnosed (1994–2013) in our institution.

## 2. Materials and Methods

### 2.1. Study Design

This was a retrospective review from January 1994 to December 2013.

### 2.2. Study Site

The study was conducted in the Department of Pathology, Korle-Bu Teaching Hospital (KBTH), the largest referral hospital in Ghana. This department receives cases from the Korle-Bu teaching hospital, hospitals within the Accra Metropolis, and the surrounding towns and Districts.

### 2.3. Data Collection and Analysis

All the histology request forms, the histology reports, and the corresponding histology slides of all thyroid malignancies diagnosed in our institution from January 1994 to December 2013 were reviewed. Data were collected on the demographic features and the histopathological characteristics of TCs diagnosed during the period of review. Data were entered into a statistical data base and analysed using SPSS software version 23.0 (Chicago) and Graph pad prism version 5.00 (https://www.graphpad.com for Windom).

The relative proportions of thyroid malignancies over the period of study were calculated.

The annual trend of follicular thyroid cancers (FTCs) over the period 1994–2013 was determined.

Descriptive statistics were computed for the ages (mean, standard deviation) of all patients included in the study.

The clinicopathological characteristics of follicular thyroid cancer including the types of surgical specimens were described. Comparisons were made between females and males using Fischer's exact test.

The histological subtypes FTC and their proportions were determined.

The results were presented in frequency tables and a histogram.

### 2.4. Availability of Data

The data used to prepared this manuscript will be made available on demand.

## 3. Inclusion Criteria

All histologically confirmed thyroid malignancies were included.

## 4. Exclusion Criteria

Poorly fixed specimens and cases with incomplete records were excluded* (a total of 3). Reasons for exclusion are as follows: one case was submitted in fragments, making it difficult to access capsular invasion, one had only the patient's name on an X-ray request form, and the last one was poorly fixed with loss of structural details*.

## 5. Results

### 5.1. Annual Distribution of Follicular Thyroid Carcinomas (1994–2013)

**Figure 1:**
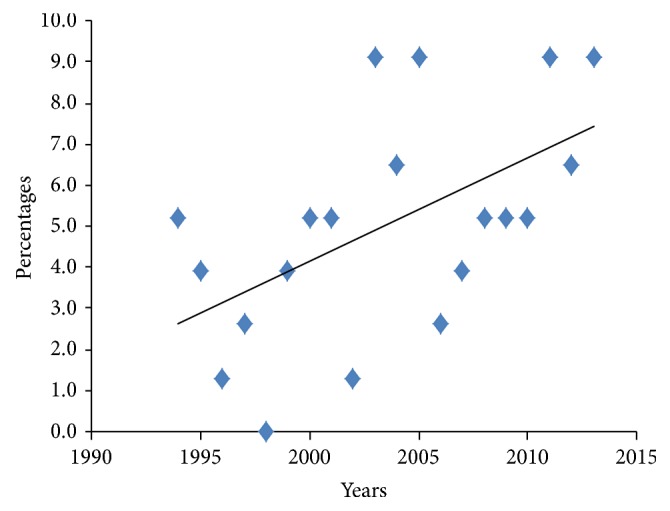
Annual Trend in FTC for the period 1994–2013.

Over the period of study, a total of 220 thyroid malignancies were diagnosed, of which 77 (35.0%) were follicular thyroid cancers (FTCs), second to papillary thyroid carcinoma 116 (52.7%).* The others were medullary 22 (10.0%) and anaplastic 5 (2.3%)*. Over the period, there was a gradual rise in the numbers of FTCs diagnosed in our institution, Figure 1.

### 5.2. Age Characteristics of Patients Diagnosed with FTC

**Figure 2:**
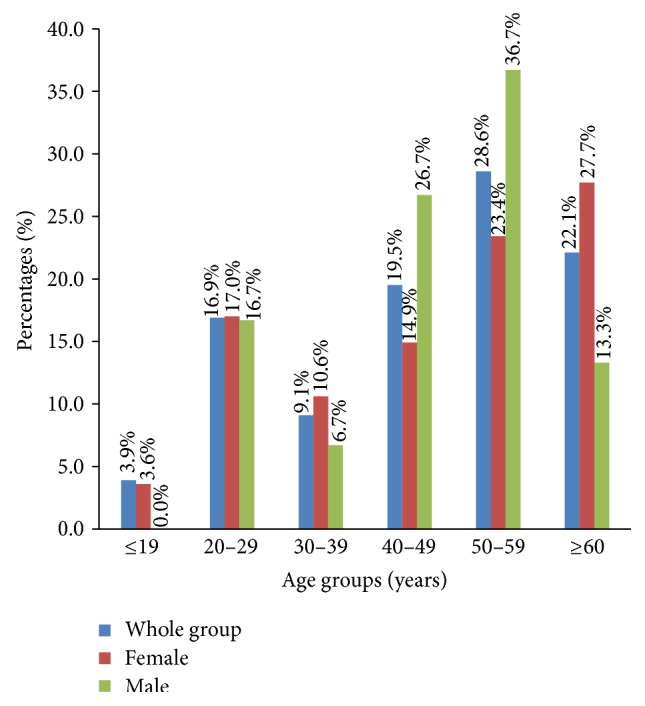
Age characteristics of patients diagnosed with thyroid cancers.

The ages of patients diagnosed with FTC range from 10 to 77 years with mean age of 46.7 years (SD = 15.7) and a modal age group of 50–59 years 22 (28.6%), Figure 2.

There were 47 (61.0%) and 30 (39.0%), with a female to male ratio of 1.5 : 1. The ages of female with FTCs ranged from 10 to 77 with a mean age of 46.9 (SD = 17.3) and a modal age group being women aged 60 years and above (27.7%), Figure 2 and Table 1. The ages of the males ranged from 21 to 66 with a mean age of 46.4 years (SD = 13.3) and a modal age group of 50–59 years (36.7%), Figure 2 and Table 1.

### 5.3. Clinical Presentation, Duration at Presentation, and Type of Surgical Specimens

The commonest symptom of TCs in both females (91.3%) and males (86.7%) was anterior neck swelling (*P* value = 0.730). The duration of symptoms at presentation was available for 31 cases of which more than half (52.7%) presented at 4 or more years followed by those who presented within one year of noticing the swelling (25.8%). A total of 22 male cases had stated duration at presentation of which many (31.8%) presented at 4 or more years, with 27.3% presenting within 3 years of onset of the disease, Table 1. In both females (46.8%) and males (46.7%), FTCs were commonly diagnosed in total thyroidectomy specimens, (*P* value = 1.000), Table 1.

### 5.4. Histological Subtypes of FTCs

*The great majority, 73 (94.8%), of the FTCs were invasive, with only 3 (3.9%) minimally invasive counterparts, Table 2*. Conventional FTC was the commonest type in both groups [females (80.9%), males (60.0%)] (*P* value = 0.066). Similarly, Hurthle cell carcinoma was the commonest variant of FTCs in this study, accounting for 10.6% in females and 26.7% in males (*P* value = 0.116), Table 2.

### 5.5. Distant Spread at the Time of Histological Diagnosis

A total of 9 (19.1%) of the female have their cancer spread outside the thyroid gland at diagnosis, mostly involving cervical lymph nodes 4 (44.4%), (*P* value = 0.633). For the males, 7 (23.3%) had distant spread at diagnosis, commonly bony involvements 4 (57.2%) (*P* value = 0.106), Table 2.

## 6. Discussion

During the period under review, follicular thyroid cancer (FTC) was the second thyroid cancer, accounting for 35.0% of all the thyroid malignancies. This is in keeping with previous studies in Ghana [3, 4], some parts of Africa [5, 6], and beyond [7, 8] that found FTC as the second common type of differentiated carcinomas of the thyroid gland. Historically, FTC has been linked to endemic iodine deficient regions of the World such as Ghana [9, 10]. The expectation of the author was that FTC would be the commonest thyroid malignancy in Ghana, in accordance with findings from similar geographical settings [11, 12]. However, this was not so. One potential explanation for the current finding is that the study was conducted in the southern part of the country where effects of iodine deficiency may have been masked by the consumption of iodine rich sea foods and thus do not give the true reflection of the picture across the country. This is more so because studies conducted in the northern parts of the Ghana more than 3 decades ago found higher incidence of iodine deficiency and increased prevalence of goiter in these areas especially along the Black Volta [10, 13].

Furthermore, the high (35.0%) relative proportions of FTC among TCs in the current study differ from the 13–17% quoted in some literature [14], but in keeping with the 48.0% found in India, an iodine deficient country, by Parikh et al. [7] and 68.0% in South Africa by Mulaudzi et al. [11].

There was a gradual rise in the incidence of FTC during the period under review. The author has no specific explanation for this trend. This may however be a reflection of the general rise in the incidence of thyroid malignancies globally in recent years [15, 16].

In this study, FTC was commoner in females with the female to male ratio of 1.5 : 1. Follicular thyroid cancers like other thyroid malignancies for an unknown reason are found by studies to be female predominant [14, 17–19], a fact that is supported by this current study. The study found that FTC was commonly (in both females and male) diagnosed in a relatively older individual similar to the findings of previous studies [3, 4, 9, 19].

The commonest clinical presentation of FTCs was an enlarged palpable anterior neck swelling (95.7%), in keeping with studies across the globe [3, 4, 9, 23]. In this study, 4.3% of the patients visited the health facilities with symptoms such as bone pain and fractures that are not primary symptoms of thyroid cancer. These were however found by examination and further investigations to result from metastatic follicular thyroid cancer. This supports studies that found that the primary presentation of FTC may be a metastatic disease [24, 25]. The study found that patients (both females and males) with FTC commonly presented late to health facilities with disease, as found in other studies [9, 23]. FTC was common in total thyroidectomy specimens as found in other studies [12, 20, 23, 26].

Conventional FTC was commoner in the females (*P* value = 0.066) while the Hurthle variant was commoner in males (*P* values = 0.116) [20]. The current study found that 20.8% of the cases have spread beyond the thyroid gland at histologic diagnosis. In descending, these were cervical lymph node, bone, neck muscles, and dura mater. Nodal involvement was commoner in females (*P* value = 0.633), while metastasis to bone was commoner in males (*P*-value = 0.106). Distant spread of FTCs at histologic diagnosis particularly to bones and brain has been reported in the literature. Our findings are thus in keeping with these previous studies [21, 22, 27]. The most likely explanation of the high proportions of cases with distant metastasis at histological diagnosis may be due to the fact that FTCs have affinity for blood vessels, a property that is considered as a key diagnostic feature.

The prognosis is general described as being favourable [27–29]. Although we have no data on the survival rate of the 35.0% people diagnosed with FTCs in this study, since there is no national cancer registry in Ghana, the author envisaged that the prognosis of the affected individuals will be favourable.

## 7. Conclusion

The study found a relative proportion of TFCs of 35.0%, with a gradual rise in the trend over the period of study. Patients presented very late with huge thyroid gland enlargement. The female to male ratio was 1.5 : 1. Approximately, 20.8% had extraglandular spread at the time of histological diagnosis.

## Figures and Tables

**Table 1:** Demographic characteristics and the clinical presentation of FCTs in females and males.

	Female (*n*/%)	Male (*n*/%)	*P* value
*Age group (years)*			
≤19	3 (6.4)	0 (0.0)	0.277
20–29	8 (17.0)	5 (16.7)	1.000
30–39	5 (10.6)	2 (6.7)	0.699
40–49	7 (14.9)	8 (26.7)	0.245
50–59	11 (23.4)	11 (36.7)	0.301
≥60	13 (27.7)	4 (13.3)	0.168
*Clinical presentation*			
Anterior neck swelling	42 (89.4)	26 (86.7)	0.730
Pathological fracture	0 (0.0)	2 (6.7)	0.149
Bone pain	2 (4.3)	1 (3.3)	1.000
Soft tissue swelling	3 (6.3)	1 (3.3)	1.000
*Duration of symptoms at presentation (years)*			
1	8 (25.8)	5 (22.7)	1.000
2	2 (6.5)	4 (18.2)	0.201
3	4 (12.9)	6 (27.3)	0.175
≥4	17 (52.7)	7 (31.8)	0.315
*Surgical specimens*			
Total thyroidectomy	22 (46.8)	14 (46.7)	1.000
Near total thyroidectomy	5 (10.6)	4 (13.3)	0.730
Lobectomy	12 (25.6)	4 (13.3)	0.256
Biopsy	8 (17.0)	7 (23.3)	0.562
Neck dissection	0 (0.0)	1 (1.3)	0.390

**Table 2:** Histological subtypes and distant spread of FTCs at the time of histological diagnosis in females and males.

	Female (*n*/%)	Male (*n*/%)	*P* value
*Histological subtype of FTC*			
Conventional	38 (80.9)	18 (60.0)	0.066
Hurthle cell	5 (10.6)	8 (26.7)	0.116
Insular	1 (2.1)	1 (3.3)	1.000
*Minimally invasive*	1 (2.1)	2 (6.7)	0.557
Encapsulated	1 (2.1)	0 (0.0)	1.000
Uncertain malignant potential	1 (2.1)	1 (3.3)	1.000
Total	47 (100.0)	30 (100.0)	
*Distant spread*	9 (19.1)	7 (23.3)	0.775
Lymph node	4 (44.4)	2 (28.6)	0.633
Bony involvements	1 (11.1)	4 (57.2)	0.106
(i) Femur	0 (0.0)	1 (14.3)	1.000
(ii) Clavicle	0 (0.0)	1 (14.3)	1.000
(iii) Cervical vertebrae	1 (11.1)	1 (14.3)	0.400
(iv) Rib	0 (0.0)	1 (14.3)	0.000
Forehead (swelling)	1 (11.1)	0 (0.0)	1.000
Neck muscle	2 (22.2)	1 (14.3)	1.000
Dural mater	1 (11.1)	0 (0.0)	1.000
Total	9 (100.0)	7 (100.0)	

## Data Availability

The data used to prepare this manuscript will be made available on demand.
